# 3,5-Difluoro­phenyl phenyl sulfone

**DOI:** 10.1107/S1600536808034302

**Published:** 2008-10-25

**Authors:** David A Grossie, Eric Fossum, Andrea Elsen, Tricia Meyer

**Affiliations:** a Department of Chemistry, Wright State University, 3640 Colonel Glenn Hwy., Dayton, Ohio 45435, USA

## Abstract

In the title compound, C_12_H_8_F_2_O_2_S, which is a precursor of functionalised poly(aryl­ene ether sulfone) polymers, the dihedral angle between the aromatic ring planes is 84.43 (8)°. In the crystal structure, aromatic π–π stacking [centroid–centroid separations = 3.808 (3) and 3.867 (3) Å] helps to establish the packing. A short C—H⋯F contact also occurs.

## Related literature

For general background, see: Attwood *et al.* (1977 ▶); Salamon (1999 ▶); Johnson *et al.* (1967 ▶); Kaiti *et al.* (2006 ▶).
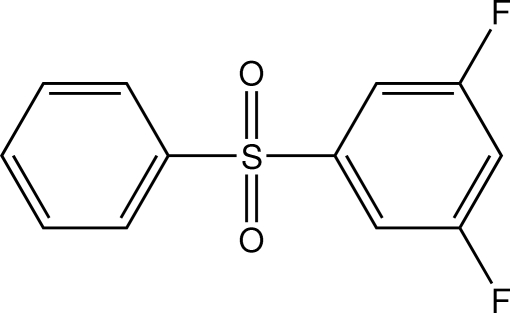


## Experimental

### 

#### Crystal data

C_12_H_8_F_2_O_2_S
*M* *_r_* = 254.24
Monoclinic, 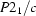

*a* = 10.328 (6) Å
*b* = 14.256 (9) Å
*c* = 7.641 (4) Å
β = 108.17 (4)°
*V* = 1068.9 (11) Å^3^
*Z* = 4
Mo *K*α radiation
μ = 0.32 mm^−1^
*T* = 173 (2) K
0.31 × 0.23 × 0.07 mm

#### Data collection

Bruker SMART APEXII CCD diffractometer
Absorption correction: multi-scan (*SADABS*; Bruker, 2003 ▶) *T*_min_ = 0.892, *T*_max_ = 0.977
9888 measured reflections
2841 independent reflections
2312 reflections with *I* > 2σ(*I*)
*R*_int_ = 0.035

#### Refinement

*R*[*F* ^2^ > 2σ(*F* ^2^)] = 0.049
*wR*(*F* ^2^) = 0.138
*S* = 1.07
2841 reflections
154 parameters
H-atom parameters constrained
Δρ_max_ = 1.03 e Å^−3^
Δρ_min_ = −0.37 e Å^−3^

### 

Data collection: *SMART* (Bruker, 2003 ▶); cell refinement: *SAINT-Plus* (Bruker, 2003 ▶); data reduction: *SAINT-Plus*; program(s) used to solve structure: *SHELXS97* (Sheldrick, 2008 ▶); program(s) used to refine structure: *SHELXL97* (Sheldrick, 2008 ▶); molecular graphics: *Mercury* (Macrae *et al.*, 2006 ▶) and *OSCAIL* (McArdle, 1995 ▶); software used to prepare material for publication: *enCIFer* (Allen *et al.* 2004 ▶) and *publCIF* (Westrip, 2008 ▶).

## Supplementary Material

Crystal structure: contains datablocks I, global. DOI: 10.1107/S1600536808034302/hb2817sup1.cif

Structure factors: contains datablocks I. DOI: 10.1107/S1600536808034302/hb2817Isup2.hkl

Additional supplementary materials:  crystallographic information; 3D view; checkCIF report

## Figures and Tables

**Table 1:** Hydrogen-bond geometry (Å, °)

*D*—H⋯*A*	*D*—H	H⋯*A*	*D*⋯*A*	*D*—H⋯*A*
C2—H2⋯F5^i^	0.95	2.44	3.337 (3)	157

Symmetry code: (i) 
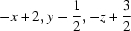
.

**Fig. 1.**
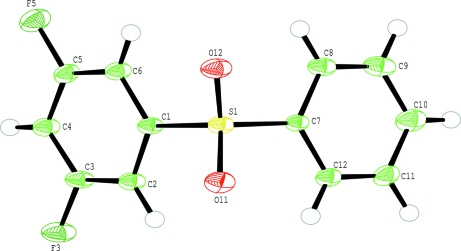
The molecular structure of (I) showing 50% displacement ellipsoids for the non-hydrogen atoms.

## supplementary crystallographic information

### Comment

Poly(arylene ether sulfone)s, PAESs, are a class of tough, amorphous polymers that possess excellent thermo and oxidative stability as well as low dielectric constants (Salamon, 1999). Several of these systems have found commercial applications that require hydrolytic and thermal stability. Classically, PAESs are synthesized through nucleophilic aromatic substitution (NAS) reactions of 4-chloro (or fluoro-) phenyl sulfone (I) with various bisphenolates, a well known A~2~ + B~2~ polycondensation, to afford linear PAESs (Attwood *et al.*, 1977, Johnson *et al.*, 1967). In order to tailor the chemical and physical properties of PAESs, it is often desirable to introduce functional groups along or pendant to the backbone. To that end, a geometric isomer of (I), the title compound, (II), has been prepared and successfully polymerized, under NAS conditions, to generate PAESs carrying a pendant phenyl sulfonyl group (Kaiti *et al.*, 2006). The pendant phenyl sulfonyl group provides a unique platform from which to access PAESs bearing a wide variety of functional groups.  We now describe the crystal structure of (II) (Fig. 1).

The bond lengths within (I) are all within their expeted ranges of values. Bond angles within the molecule were also mostly observed as expected.  The O1—S1—O2 angle is 120.39 (10)° and angles near 108° are seen for Ox—S1—Cy (with *x* = 1 or 2 and *y* = 1 or 7).  The angle between C1—S1—C7 is 102.68 (10)°, which is smaller than would have been expected, based on prediction or comparison with similar structures in CSD.

Four molecules are present within the unit cell, in two columns in which the fluorine substituted rings are stacked in the c direction with a centroid-centroid separation of 3.867 (3)Å. Neighboring columns are interconnected *via*π-π interactions between the unsubstituted phenyl rings that lie parallel to each other, separated by 3.808 (3)Å. A short C—H···F contact (Table 1) interconnects the columns within the crystal.

### Experimental

In a 250-ml round bottomed flask equipped with a stir bar, addition funnel, condenser, and gas inlet were placed 2.105 g (86.6 mmol) of Mg turnings and enough THF to cover the metal. A solution of 15.94 g (82.5 mmol) of 1-bromo-3,5-difluorobenzene and 50 ml of THF was added slowly to the stirred Mg at room temperature; upon complete addition, the reaction mixture was stirred and allowed to react for 4 h. The resulting solution of 3,5-difluorophenylmagnesium bromide was transferred to an addition funnel and added dropwise to a mixture of 16.01 g (90.8 mmol) of benzenesulfonyl chloride in 60 ml of THF at 273 K. The reaction mixture was stirred overnight. The reaction mixture was then diluted in 500 ml of ether and washed in a separatory funnel with dilute HCl, distilled water, 5% NaHCO_3_, and again with distilled H_2_O. The ether layer was dried over MgSO_4_, filtered, and then evaporated to dryness to afford a yellow solid which was recrystallized, first from ethanol/water and then from hexanes to yield colourless blocks of (I).

### Crystal data

**Table:**

C_12_H_8_F_2_O_2_S	*F*(000) = 520
*M**_r_* = 254.24	*D*_x_ = 1.580 Mg m^−^^3^
Monoclinic, *P*2_1_/*c*	Melting point: 373 K
Hall symbol: -P 2ybc	Mo *K*α radiation, λ = 0.71069 Å
*a* = 10.328 (6) Å	Cell parameters from 2499 reflections
*b* = 14.256 (9) Å	θ = 2.5–29.0°
*c* = 7.641 (4) Å	µ = 0.32 mm^−^^1^
β = 108.17 (4)°	*T* = 173 K
*V* = 1068.9 (11) Å^3^	Block, colourless
*Z* = 4	0.31 × 0.23 × 0.07 mm

### Data collection

**Table:**

Bruker SMART APEXII CCD diffractometer	2841 independent reflections
Radiation source: fine-focus sealed tube	2312 reflections with *I* > 2σ(*I*)
graphite	*R*_int_ = 0.036
ω scans	θ_max_ = 29.0°, θ_min_ = 2.1°
Absorption correction: multi-scan (*SADABS*; Bruker, 2003)	*h* = −14→14
*T*_min_ = 0.892, *T*_max_ = 0.977	*k* = −19→19
9888 measured reflections	*l* = −10→10

### Refinement

**Table:**

Refinement on *F*^2^	Primary atom site location: structure-invariant direct methods
Least-squares matrix: full	Secondary atom site location: difference Fourier map
*R*[*F*^2^ > 2σ(*F*^2^)] = 0.049	Hydrogen site location: inferred from neighbouring sites
*wR*(*F*^2^) = 0.138	H-atom parameters constrained
*S* = 1.07	*w* = 1/[σ^2^(*F*_o_^2^) + (0.0653*P*)^2^ + 0.8586*P*] where *P* = (*F*_o_^2^ + 2*F*_c_^2^)/3
2841 reflections	(Δ/σ)_max_ = 0.001
154 parameters	Δρ_max_ = 1.03 e Å^−^^3^
0 restraints	Δρ_min_ = −0.37 e Å^−^^3^

### Special details

**Table:**

Geometry. All e.s.d.'s (except the e.s.d. in the dihedral angle between two l.s. planes) are estimated using the full covariance matrix. The cell e.s.d.'s are taken into account individually in the estimation of e.s.d.'s in distances, angles and torsion angles; correlations between e.s.d.'s in cell parameters are only used when they are defined by crystal symmetry. An approximate (isotropic) treatment of cell e.s.d.'s is used for estimating e.s.d.'s involving l.s. planes.Least-squares planes (*x*,*y*,*z* in crystal coordinates) and deviations from them (* indicates atom used to define plane)0.6216 (0.0092) *x* - 0.3744 (0.0129) *y* + 7.1009 (0.0056) *z* = 6.1246 (0.0124)* -0.0042 (0.0015) C1 * 0.0030 (0.0015) C2 * 0.0017 (0.0017) C3 * -0.0051 (0.0016) C4 * 0.0041 (0.0015) C5 * 0.0006 (0.0015) C6 - 0.1026 (0.0029) S1Rms deviation of fitted atoms = 0.00357.3721 (0.0082) *x* + 9.8314 (0.0114) *y* - 2.5872 (0.0066) *z* = 9.1191 (0.0077)Angle to previous plane (with approximate e.s.d.) = 84.43 (0.08)* -0.0002 (0.0014) C7 * -0.0020 (0.0015) C8 * 0.0008 (0.0016) C9 * 0.0026 (0.0016) C10 * -0.0048 (0.0015) C11 * 0.0036 (0.0015) C12 0.1082 (0.0028) S1Rms deviation of fitted atoms = 0.0028
Refinement. Refinement of *F*^2^ against ALL reflections. The weighted *R*-factor *wR* and goodness of fit *S* are based on *F*^2^, conventional *R*-factors *R* are based on *F*, with *F* set to zero for negative *F*^2^. The threshold expression of *F*^2^ > σ(*F*^2^) is used only for calculating *R*-factors(gt) *etc*. and is not relevant to the choice of reflections for refinement. *R*-factors based on *F*^2^ are statistically about twice as large as those based on *F*, and *R*- factors based on ALL data will be even larger.

### Fractional atomic coordinates and isotropic or equivalent isotropic displacement parameters (Å^2^)

**Table:**

	*x*	*y*	*z*	*U*_iso_*/*U*_eq_	
S1	0.75260 (5)	0.58823 (4)	0.81320 (7)	0.02008 (16)	
F3	1.22708 (15)	0.66341 (11)	0.7875 (2)	0.0422 (4)	
F5	0.94947 (16)	0.91584 (10)	0.8343 (2)	0.0382 (4)	
O1	0.67321 (17)	0.62902 (12)	0.9183 (2)	0.0278 (4)	
O2	0.81161 (17)	0.49637 (11)	0.8585 (2)	0.0283 (4)	
C1	0.8867 (2)	0.66759 (14)	0.8195 (3)	0.0186 (4)	
C2	1.0092 (2)	0.63183 (15)	0.8079 (3)	0.0231 (4)	
H2	1.0244	0.5663	0.8039	0.028*	
C3	1.1074 (2)	0.69651 (16)	0.8025 (3)	0.0251 (5)	
C4	1.0896 (2)	0.79234 (15)	0.8082 (3)	0.0243 (4)	
H4	1.1588	0.8351	0.8029	0.029*	
C5	0.9666 (2)	0.82280 (15)	0.8219 (3)	0.0229 (4)	
C6	0.8626 (2)	0.76354 (15)	0.8274 (3)	0.0213 (4)	
H6	0.7786	0.7869	0.8361	0.026*	
C7	0.6541 (2)	0.58940 (14)	0.5789 (3)	0.0180 (4)	
C8	0.6894 (2)	0.53032 (15)	0.4556 (3)	0.0231 (4)	
H8	0.7620	0.4867	0.4979	0.028*	
C9	0.6162 (3)	0.53652 (16)	0.2697 (3)	0.0282 (5)	
H9	0.6389	0.4969	0.1836	0.034*	
C10	0.5105 (2)	0.60012 (17)	0.2093 (3)	0.0291 (5)	
H10	0.4613	0.6041	0.0817	0.035*	
C11	0.4758 (2)	0.65775 (17)	0.3324 (3)	0.0269 (5)	
H11	0.4022	0.7005	0.2895	0.032*	
C12	0.5479 (2)	0.65361 (15)	0.5189 (3)	0.0227 (4)	
H12	0.5252	0.6939	0.6041	0.027*	

### Atomic displacement parameters (Å^2^)

**Table:**

	*U*^11^	*U*^22^	*U*^33^	*U*^12^	*U*^13^	*U*^23^
S1	0.0243 (3)	0.0206 (3)	0.0158 (3)	−0.00311 (19)	0.0069 (2)	0.00231 (19)
F3	0.0273 (8)	0.0336 (8)	0.0690 (12)	0.0042 (6)	0.0201 (8)	−0.0013 (8)
F5	0.0413 (9)	0.0185 (7)	0.0601 (11)	−0.0006 (6)	0.0236 (8)	−0.0012 (6)
O1	0.0335 (9)	0.0347 (9)	0.0195 (8)	−0.0071 (7)	0.0144 (7)	−0.0025 (7)
O2	0.0316 (8)	0.0224 (8)	0.0273 (8)	−0.0026 (7)	0.0041 (7)	0.0089 (6)
C1	0.0217 (10)	0.0199 (9)	0.0141 (9)	−0.0014 (8)	0.0054 (8)	0.0014 (7)
C2	0.0240 (10)	0.0188 (10)	0.0255 (11)	0.0013 (8)	0.0063 (9)	0.0018 (8)
C3	0.0188 (10)	0.0264 (11)	0.0300 (12)	0.0022 (8)	0.0073 (9)	0.0004 (9)
C4	0.0249 (10)	0.0223 (10)	0.0266 (11)	−0.0053 (8)	0.0092 (9)	−0.0017 (9)
C5	0.0294 (11)	0.0166 (9)	0.0225 (11)	−0.0011 (8)	0.0077 (9)	−0.0019 (8)
C6	0.0247 (10)	0.0207 (10)	0.0194 (10)	0.0000 (8)	0.0081 (8)	−0.0008 (8)
C7	0.0192 (9)	0.0188 (9)	0.0171 (9)	−0.0038 (7)	0.0072 (8)	0.0007 (7)
C8	0.0285 (11)	0.0197 (10)	0.0231 (11)	−0.0009 (8)	0.0111 (9)	−0.0005 (8)
C9	0.0392 (13)	0.0261 (11)	0.0220 (11)	−0.0087 (9)	0.0135 (10)	−0.0059 (9)
C10	0.0302 (12)	0.0358 (13)	0.0179 (10)	−0.0131 (10)	0.0024 (9)	0.0017 (9)
C11	0.0208 (10)	0.0313 (12)	0.0279 (12)	−0.0029 (9)	0.0066 (9)	0.0080 (9)
C12	0.0228 (10)	0.0241 (10)	0.0237 (11)	0.0010 (8)	0.0110 (8)	0.0017 (8)

### Geometric parameters (Å, °)

**Table:**

S1—O1	1.4375 (18)	C5—C6	1.378 (3)
S1—O2	1.4403 (18)	C6—H6	0.9500
S1—C7	1.762 (2)	C7—C12	1.392 (3)
S1—C1	1.777 (2)	C7—C8	1.394 (3)
F3—C3	1.361 (3)	C8—C9	1.388 (3)
F5—C5	1.346 (3)	C8—H8	0.9500
C1—C2	1.392 (3)	C9—C10	1.383 (4)
C1—C6	1.395 (3)	C9—H9	0.9500
C2—C3	1.381 (3)	C10—C11	1.378 (4)
C2—H2	0.9500	C10—H10	0.9500
C3—C4	1.381 (3)	C11—C12	1.388 (3)
C4—C5	1.377 (3)	C11—H11	0.9500
C4—H4	0.9500	C12—H12	0.9500
			
O1—S1—O2	120.39 (10)	C5—C6—C1	116.6 (2)
O1—S1—C7	108.34 (11)	C5—C6—H6	121.7
O2—S1—C7	108.65 (10)	C1—C6—H6	121.7
O1—S1—C1	107.63 (10)	C12—C7—C8	121.3 (2)
O2—S1—C1	107.72 (11)	C12—C7—S1	119.13 (16)
C7—S1—C1	102.68 (10)	C8—C7—S1	119.42 (17)
C2—C1—C6	122.68 (19)	C9—C8—C7	118.7 (2)
C2—C1—S1	118.76 (16)	C9—C8—H8	120.7
C6—C1—S1	118.50 (16)	C7—C8—H8	120.7
C3—C2—C1	116.6 (2)	C10—C9—C8	120.3 (2)
C3—C2—H2	121.7	C10—C9—H9	119.9
C1—C2—H2	121.7	C8—C9—H9	119.9
F3—C3—C4	118.6 (2)	C11—C10—C9	120.6 (2)
F3—C3—C2	117.8 (2)	C11—C10—H10	119.7
C4—C3—C2	123.6 (2)	C9—C10—H10	119.7
C5—C4—C3	116.7 (2)	C10—C11—C12	120.4 (2)
C5—C4—H4	121.7	C10—C11—H11	119.8
C3—C4—H4	121.7	C12—C11—H11	119.8
F5—C5—C4	117.46 (19)	C11—C12—C7	118.8 (2)
F5—C5—C6	118.8 (2)	C11—C12—H12	120.6
C4—C5—C6	123.8 (2)	C7—C12—H12	120.6
			
O1—S1—C1—C2	150.34 (17)	C2—C1—C6—C5	0.4 (3)
O2—S1—C1—C2	19.1 (2)	S1—C1—C6—C5	−176.56 (16)
C7—S1—C1—C2	−95.46 (18)	O1—S1—C7—C12	22.96 (19)
O1—S1—C1—C6	−32.6 (2)	O2—S1—C7—C12	155.37 (16)
O2—S1—C1—C6	−163.77 (16)	C1—S1—C7—C12	−90.72 (18)
C7—S1—C1—C6	81.64 (18)	O1—S1—C7—C8	−161.09 (16)
C6—C1—C2—C3	−0.6 (3)	O2—S1—C7—C8	−28.68 (19)
S1—C1—C2—C3	176.34 (17)	C1—S1—C7—C8	85.23 (18)
C1—C2—C3—F3	−178.7 (2)	C12—C7—C8—C9	0.0 (3)
C1—C2—C3—C4	0.1 (3)	S1—C7—C8—C9	−175.82 (16)
F3—C3—C4—C5	179.5 (2)	C7—C8—C9—C10	−0.1 (3)
C2—C3—C4—C5	0.7 (4)	C8—C9—C10—C11	−0.3 (3)
C3—C4—C5—F5	177.6 (2)	C9—C10—C11—C12	0.9 (3)
C3—C4—C5—C6	−0.9 (3)	C10—C11—C12—C7	−0.9 (3)
F5—C5—C6—C1	−178.16 (19)	C8—C7—C12—C11	0.5 (3)
C4—C5—C6—C1	0.4 (3)	S1—C7—C12—C11	176.35 (16)

### Hydrogen-bond geometry (Å, °)

**Table:**

*D*—H···*A*	*D*—H	H···*A*	*D*···*A*	*D*—H···*A*
C2—H2···F5^i^	0.95	2.44	3.337 (3)	157

Symmetry codes: (i) −*x*+2, *y*−1/2, −*z*+3/2.

## References

[bb1] Allen, F. H., Johnson, O., Shields, G. P., Smith, B. R. & Towler, M. (2004). *J. Appl. Cryst.* **37**, 335–338.

[bb2] Attwood, T. E., Barr, D. A., Feasey, G. G., Leslie, V. J., Newton, A. B. & Rose, J. B. (1977). *Polymer* **18**, 354–358.

[bb3] Bruker (2003). *SMART* and *SAINT-Plus* Bruker AXS Inc., Madison, Wisconsin, USA.

[bb4] Johnson, R. N., Farnham, A. G., Clendinning, R., Hale, W. F. & Merriam, C. N. (1967). *J. Polym. Sci. Part. A Polym. Chem.* **5**, 2375–2398.

[bb5] Kaiti, S., Himmelberg, P., Williams, J., Abdellatif, M. & Fossum, E. (2006). *Macromolecules*, **39**, 7909–7914.

[bb6] Macrae, C. F., Edgington, P. R., McCabe, P., Pidcock, E., Shields, G. P., Taylor, R., Towler, M. & van de Streek, J. (2006). *J. Appl. Cryst.* **39**, 453–457.

[bb7] McArdle, P. (1995). *J. Appl. Cryst.* **28**, 65. 10.1107/S1600576721008529 PMC8493623 34667454

[bb8] Salamon, J. C. (1999). Editor. *Concise Polymeric Materials Encyclopedia* Boca Raton: CRC Press LLC.

[bb9] Sheldrick, G. M. (2008). *Acta Cryst.* A**64**, 112–122. 10.1107/S0108767307043930 18156677

[bb11] Westrip, S. P. (2008). *publCIF* In preparation.

